# Epidemiology of Post-traumatic Stress Disorder in Iranian Population From 2019 to 2024: A Systematic Review and Meta-analysis

**DOI:** 10.34172/aim.31230

**Published:** 2024-10-01

**Authors:** Asad Imani, Shahram Molavynejad, Mojgan Khademi, Mohammad Adineh, Elham Shafiei, Mohsen Savaie

**Affiliations:** ^1^Student Research Committee, School of Nursing and Midwifery, Ahvaz Jundishapur University of Medical Sciences, Ahvaz, Iran; ^2^Nursing Care Research Center in Chronic Diseases, School of Nursing and Midwifery, Ahvaz Jundishapur University of Medical Sciences, Ahvaz, Iran; ^3^Social Determinants of Health Research Center, School of Nursing and Midwifery, Lorestan University of Medical Sciences, Khorramabad, Iran; ^4^Psychosocial Injuries Research Center, Ilam University of Medical Sciences, Ilam, Iran; ^5^Pain Research Center, Ahvaz Jundishapur University of Medical Sciences, Ahvaz, Iran

**Keywords:** Iran, Post-traumatic stress disorder, Prevalence, Trauma

## Abstract

**Background:**

Post-traumatic stress disorder (PTSD) is a disorder that arises from experiencing traumatic events such as traffic accidents, war, natural disorders, and job incidents. This study focused on determining the epidemiology of PTSD in the Iranian population from 2019 to 2024.

**Methods:**

In this systematic review and meta-analysis, we explored databases such as PubMed, Embase, Web of Knowledge, Scopus, and Magiran to achieve a maximum variety of screened articles. The quality of the included articles was evaluated using the STROBE checklist. For data analysis, due to the variation in reporting the PTSD prevalence across the reviewed articles, heterogeneity was assessed using the I^2^ index, and a random effect model was applied to account for this variation.

**Results:**

Out of 800 articles found in the initial review, only 15 articles were entered in the final analysis based on inclusion and exclusion criteria, with a total of 9868 participants. The overall PTSD prevalence in the Iranian population was 31.87% (95% confidence interval [CI]=17.87- 45.87, I^2^=95.29%, *P*<0.001). Additionally, PTSD prevalence in men (36.64%) was higher than in women (35.52%).

**Conclusion:**

The prevalence of PTSD in young Iranian men is relatively high, and there was no statistically significant decrease in PTSD prevalence between 2019 and 2024.

## Introduction

 Trauma has been one of the leading causes of disability and death in recent decades.^1^ A study on the prevalence of trauma among 24 European countries found that 70.4% of people suffered one or more major trauma during the year.^2^ The prevalence of trauma in Iran has been reported in various studies, ranging between 61.70%^3^ to 89.2%,^4^ making it the second leading cause of death.^5^ Iran is prone to events such as earthquakes, wars, road traffic accidents, and floods.^6^ Given the relatively high incidence of trauma in Iran and the psychosocial responses such as post-traumatic stress disorder (PTSD), it seems that investigating this phenomenon in research studies should always be considered.^7^

 PTSD is associated with work-related problems, lower quality of life, functional impairments, and physical health problems.^8^ Individuals with PTSD cases may face a prolonged recovery process, with about 10 % of individuals experiencing long-term psychological effects and chronic PTSD.^9^ A study in the United States revealed that the prevalence of PTSD one year after trauma ranged from 2.3% to 9.1%, with a lifetime prevalence from 3.4% to 26.9%.^10^ A study showed that the patterns of trauma in Iran and individual responses such as PTSD are constantly evolving.^11^ In Iran, different studies have investigated PTSD in relation to job-related trauma,^12-16^ accidents,^3^ wars,^17^ floods,^18,19^ the COVID-19,^20-22^ and childbirth.^23,24^ All these studies were conducted between 2019 and 2024.

 Since the prevalence of stressful events varies across different periods, access to updated statistics in the form of a meta-analytic study will undoubtedly help to understand the true significance of the crisis. However, these epidemiological studies were conducted locally with limited sample sizes, making them insufficient for providing clear and useful information for high-level health decision-making. Health decision-makers cannot make decisions based on small and localized studies,^25^ nor can they generalize the prevalence of PTSD found in such studies to the entire Iranian society.^26^ Health authorities need to be aware of the actual prevalence of PTSD to develop an accurate crisis control map and effective operational plans. This is possible only through a national study or a meta-analysis of multiple studies. Therefore, a systematic review and meta-analysis of PTSD research can provide useful and concise information to health decision-makers for the management and prevention of this disorder. Thus, our primary objective was to conduct a study on the prevalence of PTSD in Iran from 2019 to 2024.

## Materials and Methods

###  Search Strategies

 Due to the broad scope of trauma-related topics and the variety of studies, we searched databases of PubMed, MEDLINE via OVID, Web of Knowledge, Wiley, Scopus, Magiran, SID, and Google Scholar with the keywords, including trauma, trauma in Iran, stress disorder, post-traumatic stress disorder, post-traumatic neuroses, and chronic post-traumatic stress disorder.

 Moreover, to find articles related to the topic, we used combinations of Job and PTSD, Trauma and PTSD, War and PTSD, Earthquake and PTSD, Flood and PTSD. Other Boolean operators were used such as Trauma OR Event, Trauma OR Incident, Trauma Prevalence OR Trauma Incidence, PTSD NOT Anxiety, and PTSD NOT Stress. The searches in Google Scholar, Magiran, and SID were conducted only using the main keywords because they were not sensitive to the Boolean operators. However, searches in the other databases were carried out by combining words with Boolean operators.

###  Inclusion and Exclusion Criteria

####  Inclusion Criteria

- All subjects exposed to traumatic events (incidents causing physical, emotional, spiritual, or psychological harm) such as wars, earthquakes, job-related stress, childbirth, and floods were included.
- Articles carried out on Iranian populations.
- Articles published in Persian or English.
- Articles published between 2019 and 2024 (New statistics are always a better basis for health decision-makers. On the other hand, this period of time coincided with the COVID-19 pandemic, allowing us to investigate its effect on the prevalence of PTSD in Iran).

####  Exclusion Criteria

- Full-text not available.
- Articles published with qualitative approaches, case reports, letters to editors, and interventional studies (to maintain a quantitative approach and cohesiveness in the final analysis).

###  Qualification of Studies

 The quality of studies was evaluated using the standard 22-item STROBE checklist. The checklist items are related to the issue and abstract (question 1), introduction (questions 2 and 3), methodology (questions 4-12), results (questions 13-17), discussion (questions 18-21), and information on funding (question 22).^27^ The questions mentioned in the pertinent section of the article were awarded one point if present, or zero if absent. According to consensus among the research team, articles that achieved 75% of the total score (16 points) were included in the meta-analysis.

###  Data Extraction

 The abstracts and titles of the papers were independently reviewed by two independent reviewers according to the inclusion and exclusion criteria. Any cases of disagreement were resolved through discussion, and if necessary, all researchers reached a consensus to handle remarkable disagreements. Then, articles that met the inclusion criteria were categorized by study characteristics, including author, study location, year of publication, study method, events, overall PTSD prevalence, age range, PTSD prevalence by gender, and study population.

###  Statistical Analysis

 The main objective of this study was to determine the prevalence of overall PTSD, as well as prevalence in men and women. Although examining heterogeneity in prevalence studies is not mandatory, it was investigated in the present study due to the variation in reported prevalence levels across the reviewed studies. The I^2^ index was employed to assess the heterogeneity of the studies, with values of < 25%, 25-75%, and > 75% representing low, moderate, and high heterogeneity, respectively.

 One of the most effective approaches to deal with statistical heterogeneity in studies is to investigate the underlying causes by performing metaregression. In this study, metaregression analysis was conducted using STATA (v.17) to examine the relationship between PTSD prevalence and the year of study, study location, and event type.

## Results

**Figure 1:**
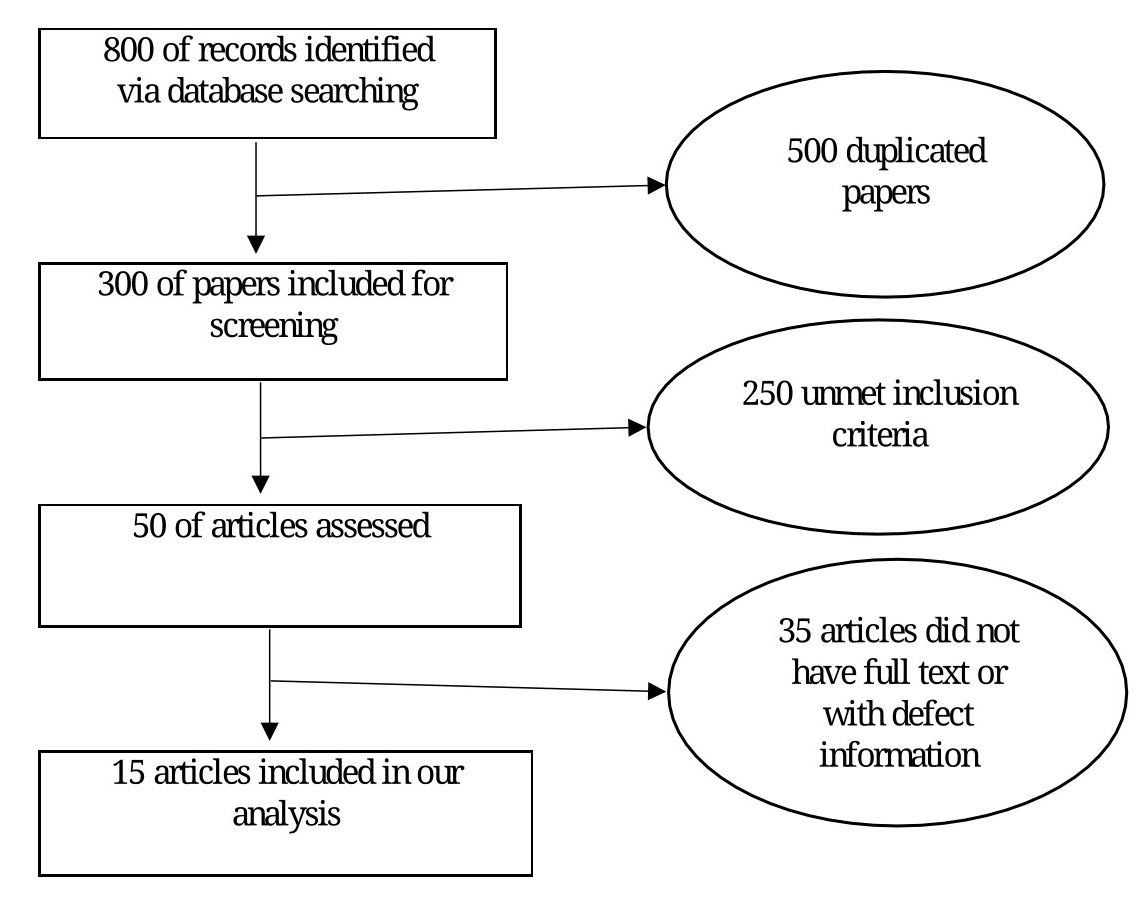
Systematic Literature Review Process

 In the initial search, 800 articles were identified, and after reviewing their titles, only 300 were considered for screening. Among these, 250 articles failed to meet the inclusion criteria for further review. Eventually, 15 articles were included for meta-analysis and systematic review despite their full texts were accessible and contained sufficient information (Figure 1).

 All studies were conducted on the Iranian population between 2019 and 2024. Table 1 summarizes the key features of the reviewed articles.

**Table 1:** Study Features

**Authors**	**Year**	**Study Type**	**Place**	**Event**	**Sample Size**	**Age (Mean±SD)**	**Overall PTSD (%)**	**Mean (%)**	**Women (%)**
Abdollahzadeh et al^28^	2023	Cross-sectional study	Mahabad	Genital mutilation	155	31.46 ± 6.94	3.9%	-	3.9%
Shabani et al^19^	2024	Cross-sectional study	Lorestan, Khouzestan, Golestan	Flood	2305	36.8 ± 12.1	24.8%	21.6%	28%
Ahmadnejad et al^23^	2021	Cross-sectional study	Baneh	Childbirth	365	28.0 ± 4.3	47.7%	-	47.7%
Hajizadeh et a^24^	2021	Prospective study	Tabriz	Childbirth	288	26.0 ± 5.3	16.3%	-	16.3%
Ebrahimi et al^13^	2021	Cross-sectional study	Shahroud	Job	228	34.03 ± 7.7	85.5%	83.3%	%87.7
Hosseininejad et al^12^	2019	Cross-sectional study	Mazandaran	Job	131	24.97 ± 2.6	82.41%	82.96%	82.08%
Hosseini et al^20^	2022	Cross-sectional study	Sari	COVID- 19	199	27.0 ± 4.3	19.1%	0.5%	18.6%
Bastami et al^18^	2024	Descriptive-analytical study	Lorestan	Flood	470	32.25 ± 6.02	23.75%	13.64%	33.9%
Keyhani et al^14^	2023	Cross-sectional study	Tehran	Job	365	34.06 ± 7.78	14.6%	14.8%	14.4%
Rostamizadeh et al^17^	2020	Retrospective cohort study	Ilam	War	227	66.67 ± 7.16	16.3%	14.3%	18.3%
Sehat et al^3^	2020	Cross-sectional population-based study	Kashan	Trauma	3880	30 ± 9	18.65%	16%	21.3%
Khademhamzehei et al^21^	2023	Cross-sectional study	Hamadan	COVID- 19	185	38.43 ± 14.07	63.5%	63.8	63.2
Khazaei et al^15^	2021	Cross-sectional study	Hamadan	Job	259	28.88 ± 6.94	22%	22%	-
Sahebi et al^16^	2020	Systematic review and meta-analysis	Iran	Job	274	35.21 ± 5.24	23.17%	23.17%	-
Faramarzi et al^22^	2023	Cross-sectional study	Babol	COVID-19	477	60.5 ± 17.9	8.1%	-	-

*Note.* SD: Standard deviation; PTSD: Post-traumatic stress disorder.

**Figure 2:**
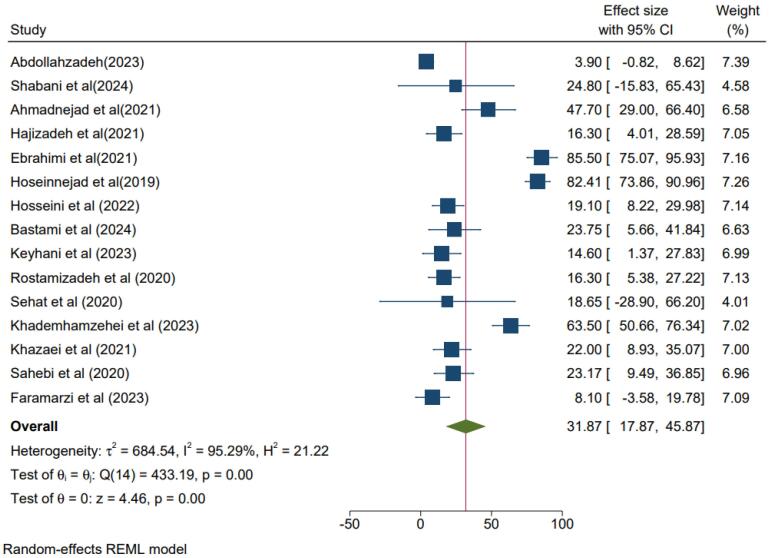
Prevalence of Overall Post-traumatic Stress Disorder With a 95% Confidence Interval

 Results indicate that the total sample size was 9868 participants, with a mean age of 35.61 ± 7.8 years. In this study, the I^2^ index for heterogeneity was 95.29. The meta-analysis was carried out through the random effects model due to the high and significant heterogeneity among the studies. According to the data analysis, the overall prevalence of PTSD was 31.87% (95% CI = 17.87- 45.87, I^2^ = 95.29%, *P*< 0.001) (Figure 2).

 Based on geographical region, the highest prevalence of PTSD was observed in Shahrood, Mazandaran, and Hamadan, respectively, which are located in different geographic regions of Iran, from north to east and west.

**Figure 3:**
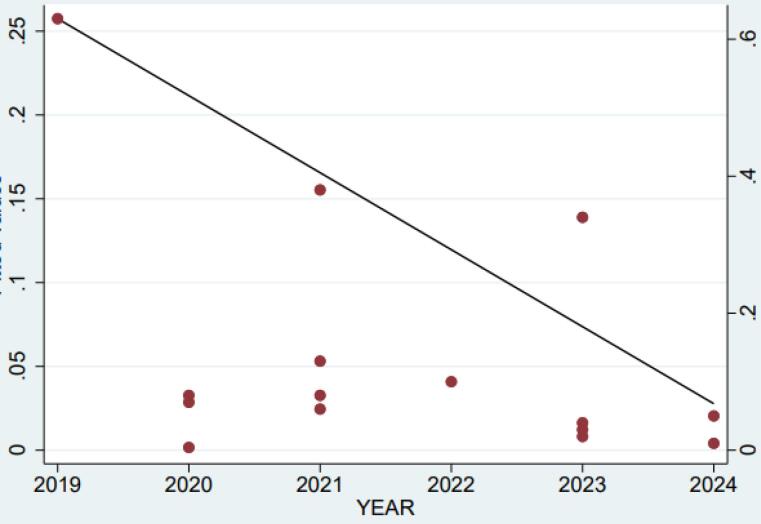
Meta-Regression Analysis Based on the Year of Publication

 As seen in Figure 3, there is a significant relationship between PTSD prevalence in Iran and the year of the study (*P* = 0.04). Furthermore, although the prevalence of PTSD in Iran has decreased from 2019 to 2024, the change was not statistically significant. Additionally, metaregression results indicated no significant association between PTSD prevalence and study location or event type (*P* > 0.05).

**Figure 4:**
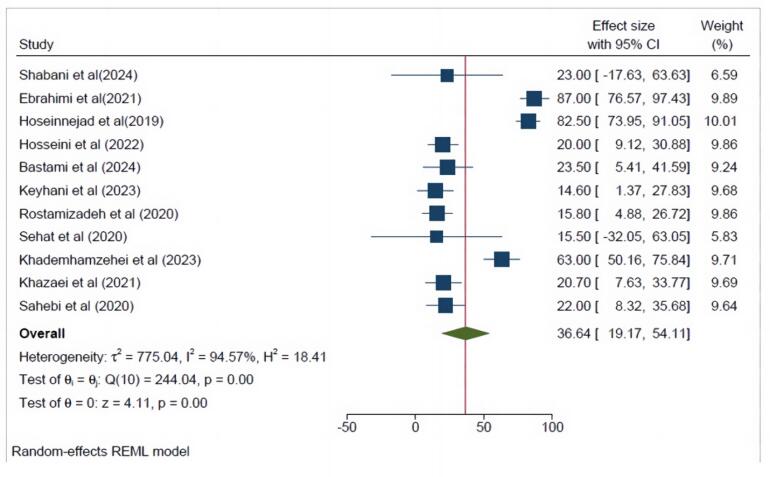
Prevalence of Post-traumatic Stress Disorder in Men With 95% Confidence Interval

**Figure 5:**
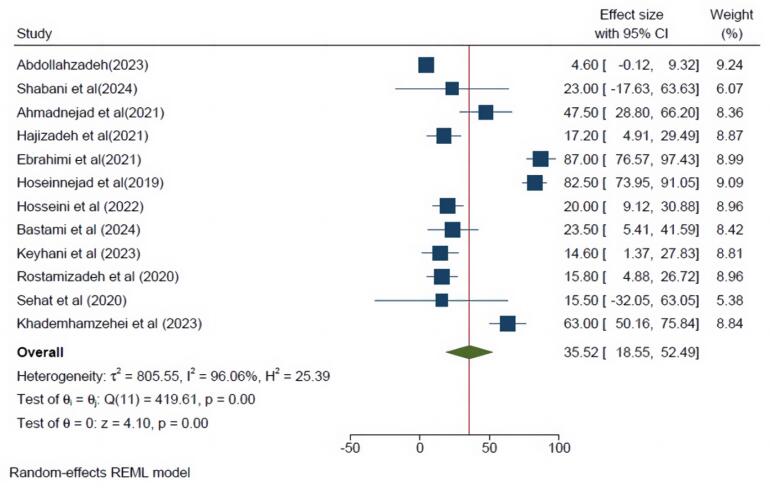
Prevalence of Post-traumatic Stress Disorder in Women With 95% Confidence Interval

Figures 4 and 5 show that the PTSD prevalence among Iranian men and women was 36.64% (95% CI = 19.17- 54.11, I^2^ = 94.57%) and 35.52% (95% CI = 18.55- 52.49, I^2^ = 96.06%), respectively.

## Discussion

 PTSD has been recognized as one of the most significant psychosocial consequences of trauma.^29^ This disorder affects the individual, social, and emotional dimensions of traumatic patients.^30^ Based on DSM-5, PTSD is categorized as “a disorder associated with trauma and stress, emerging following exposure to a traumatic event.” It is characterized by acute or chronic patterns.^31^

 Various studies have reported the prevalence of PTSD in general populations in countries such as China,^32^ South Korea,^33^ Ethiopia,^34^ Lebanon,^35^ the USA,^10^ and England^36^; These figures were determined to be 53.2%, 26.8%, 37.3%, 33.3%, 26.9%, and 7.8%, respectively. Our study found that the overall PTSD prevalence in the Iranian population was 31.87%. These heterogeneous results may stem from different study methodologies, sample sizes, and the contextual personalities of participants. Additionally, the diversity in political, social, and cultural situations across countries may contribute to this discrepancy in the prevalence rates of PTSD.

 Our meta-regression results indicated that the prevalence of PTSD in Iran has not decreased significantly from 2019 to 2024. Similar to other world countries, Iran experienced the COVID-19 pandemic during the last 5 years, which likely affected the prevalence of PTSD. Zhang et al^37^ found that PTSD prevalence during the COVID-19 pandemic in the general population was 15%. Another study in China reported that the PTSD prevalence is 4.6% in the general population and 25% in suspected or confirmed COVID-19 cases.^38^ Cook et al^39^ showed a PTSD prevalence of 23.88% during the COVID-19 pandemic. Furthermore, the results of a meta-analysis and systematic review conducted across 24 countries revealed that the overall PTSD prevalence in the general population is 17.52%.^40^

 Initially, it was expected that the occurrence of a pandemic and its association with stressful events would intensify the level of PTSD. However, the existence of contradictory findings indicates that the prevalence of PTSD is not only dependent on the pandemic but is also influenced by other factors that need to be investigated in future studies.

 The samples’ mean age was 35.61 ± 7.8 years, indicating a relatively young age. This finding is consistent with the studies by Scheeringa et al,^41^ Bastien et al,^42^ and Pasha et al.^43^ Young people are more involved in harmful behaviors and experience traumatic events than other age groups.

 Another meta-analysis finding from our study reported that the prevalence of PTSD in Iranian men (36.64%) is higher than in women (35.52%). This finding contradicts previous Iranian studies by Merghati Khoei et al,^44^ Sepahvand et al,^45^ and Rafiey et al.^46^ These contradictory findings in Iranian studies highlight the need for conducting etiological studies in the future. Nevertheless, it is clear that Iranian men are more frequently exposed to unwanted events such as war, occupational incidents, and road traffic victims.

## Conclusion

 The main objective of our meta-analysis was to determine the prevalence of PTSD in the Iranian population from 2019 to 2024, which was found to be 31.87%, representing a relatively high level. Given the occurrence of natural disasters such as earthquakes, floods, and storms in Iran every year, alongside the high incidence of road traffic accidents, it is recommended that future studies assess PTSD prevalence and the trend of its changes over time. Unlike previous studies, our findings indicated that the prevalence of PTSD is higher among young Iranian men. Hence, social organizations and healthcare policymakers should focus on this age group.
